# Real-Time Detection and Classification of *Scirtothrips dorsalis* on Fruit Crops with Smartphone-Based Deep Learning System: Preliminary Results

**DOI:** 10.3390/insects14060523

**Published:** 2023-06-05

**Authors:** Gildas Niyigena, Sangjun Lee, Soonhwa Kwon, Daebin Song, Byoung-Kwan Cho

**Affiliations:** 1 Department of Smart Agricultural System, Chungnam National University, Daejeon 34134, Republic of Korea; gildniy05@gmail.com; 2 Department of Biosystems Machinery Engineering, Chungnam National University, Daejeon 34134, Republic of Korea; sangjoon10005@naver.com; 3 Citrus Research Institute, Seogwipo 63607, Republic of Korea; shkwonn@korea.kr; 4 Department of Biosystem Bio-Industrial Machinery Engineering, Gyeongsang National University, Jinju 52828, Republic of Korea; dbsong@gnu.ac.kr

**Keywords:** *Scirtothrips dorsalis*, real time, smartphone application, lighting conditions, object detection

## Abstract

**Simple Summary:**

This study developed a real-time thrips detection application to classify the *Scirtothrips dorsalis* from other thrips species, and was optimized for working on embedded devices, such as smartphones. The performances of several deep learning models, including YOLOv5, Faster R-CNN, SSD MobileNetV2, and EfficientDet-D0, were evaluated based on their accuracy and speed in detecting thrips on yellow sticky traps. The models were trained and tested on two datasets containing thrips and non-thrips insects captured under different lighting conditions (reflectance, transmittance, and reflectance + transmittance). The developed application used the EfficientDet-D0 model trained on the transmittance dataset to detect and visualize the presence of thrips in real-time, making it a valuable tool for the monitoring and management of thrips in agriculture, with practical implications for pest control and crop yield improvement.

**Abstract:**

This study proposes a deep-learning-based system for detecting and classifying *Scirtothrips dorsalis* Hood, a highly invasive insect pest that causes significant economic losses to fruit crops worldwide. The system uses yellow sticky traps and a deep learning model to detect the presence of thrips in real time, allowing farmers to take prompt action to prevent the spread of the pest. To achieve this, several deep learning models are evaluated, including YOLOv5, Faster R-CNN, SSD MobileNetV2, and EfficientDet-D0. EfficientDet-D0 was integrated into the proposed smartphone application for mobility and usage in the absence of Internet coverage because of its smaller model size, fast inference time, and reasonable performance on the relevant dataset. This model was tested on two datasets, in which thrips and non-thrips insects were captured under different lighting conditions. The system installation took up 13.5 MB of the device’s internal memory and achieved an inference time of 76 ms with an accuracy of 93.3%. Additionally, this study investigated the impact of lighting conditions on the performance of the model, which led to the development of a transmittance lighting setup to improve the accuracy of the detection system. The proposed system is a cost-effective and efficient alternative to traditional detection methods and provides significant benefits to fruit farmers and the related ecosystem.

## 1. Introduction

*Scirtothrips dorsalis* Hood, a species of the order Thysanoptera, is a highly invasive pest that causes significant economic losses in fruit crops worldwide [1]. *Scirtothrips dorsalis* is native to Southern Asia [2] but has subsequently spread to Northern Asia and Australia, as well as several countries in America, Europe, and Africa, causing severe damage to various crops, such as mangoes, citrus, and grapes [3]. This polyphagous pest feeds on the flowers, leaves, and fruits of host plants and causes premature leaf fall, fruit deformation, and reduced fruit quality. It is a vector for tospoviruses, such as Tomato spotted wilt virus [4], which cause necrosis, chlorosis, and yellowing of plant tissues, leading to significant yield losses [5].

*Scirtothrips dorsalis* infections have been a significant problem in Asia for over a decade, with outbreaks reported in China, India, Thailand, and Vietnam [1]. *Scirtothrips dorsalis* was identified as a new crop pest in South Korea in 2007 [6]. This pest causes substantial damage to crops, especially strawberries, grapes, blueberries, and citrus orchards, which are important fruit crops in South Korea [7].

Currently, the detection and identification of *S. dorsalis* is challenging, time-consuming, and expensive. Traditional methods, such as visual inspection and sticky traps, are ineffective, especially in cases of low population densities or hidden infestations. Chemical control is also limited because *S. dorsalis* has developed resistance to several insecticides [8] and the use of broad-spectrum pesticides can harm beneficial insects and disrupt ecosystems [9].

In recent years, there has been an increasing interest in utilizing deep learning systems for the detection and classification of insect pests [10]. These systems employ artificial neural networks that learn from extensive data and identify patterns that are challenging for humans to discern [11]. They can be trained to differentiate between various species, insect life stages, and healthy/infested plants, thereby enabling real-time and accurate detection [12]. For example, a supervised artificial neural network (ANN) model was developed to semi-automatically identify eighteen common European species of Thysanoptera from four genera. The ANN utilized morphometric and qualitative character data from different body parts, along with sex information, achieving a 97% accurate identification of both male and female specimens in an independent test. These findings highlight the potential of ANN for wider application in Thysanoptera identification practices [13]. Furthermore, according to P. Fedor et al., the artificial neural networks have proven effective in accurately distinguishing between two closely related species, *T. sambuci* and *T. fuscipennis*, despite their similar morphology. This demonstrates the approach’s reliability and speed in species identification, making it a valuable tool for online, semi-automated pest identification [14].

The objective of this study was to create a system to identify thrips using smartphones. The system uses yellow sticky traps obtained from fruit farms and is equipped with a deep learning model that enables it to function in areas without Internet coverage. The system was designed to detect the presence of thrips insects in real-time at an early stage of occurrence, thus allowing farmers to take prompt action to prevent the spread of the pest. This system is a cost-effective and efficient alternative to traditional detection methods and provides significant benefits to fruit farmers and overall ecosystems.

To achieve this goal, several deep learning models, namely, YOLOv5, Faster R-CNN, SSD MobileNetV2, and EfficientDet-D0, were evaluated based on their accuracy and speed in detecting thrips on yellow sticky traps. The models were trained and tested on two datasets containing thrips and non-thrips insects captured under different lighting conditions. The best model was integrated into the proposed application. Moreover, the impact of lighting conditions on the performance of the model was investigated, leading to the development of a lighting setup to improve the accuracy of the detection system.

## 2. Materials and Methods

### 2.1. Data Collection

#### Sticky Trap Cards and Insect Position Marking

**Figure 1:**
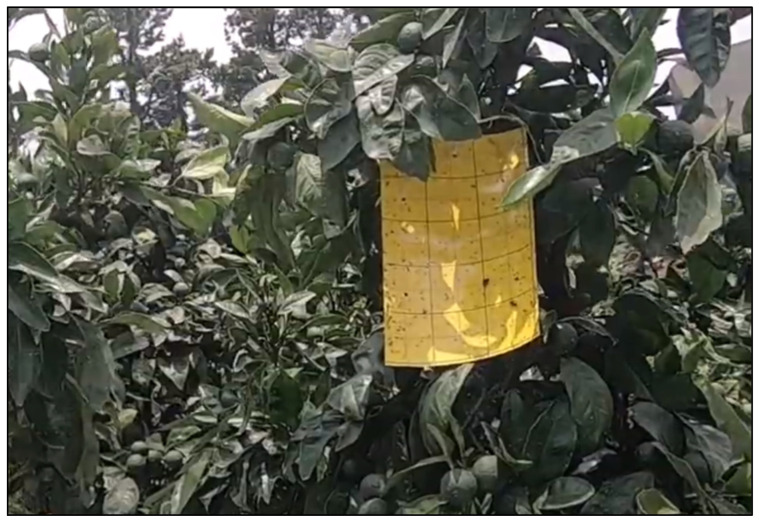
Yellow sticky trap card placed in the citrus orchard experimental farm.

In the summer of 2021, a total of 65 sticky traps were placed in citrus fruit tree orchards (Figure 1) at the experimental farm of the National Institute of Horticultural and Herbal Science, Rural Development Administration, located on Jeju Island in South Korea. The sticky traps were manufactured by KIP Inc, a Korean company, and were made of bright yellow plastic cards measuring 150 × 250 mm each. The yellow color of the traps makes them attractive to a wide range of insects, including thrips [15], which are most active during the summer months [16]. To effectively trap thrips, both sides of the traps were coated with a sticky adhesive. The traps were hung from the branches of fruit trees using a wired string at a height of 1.5 to 2 m above the ground (easily visible to thrips, and typically at human eye level), with a density of 5 to 10 traps per acre. One side of the trap was left uncovered to capture the specimens and the other side was covered with white paper. The traps were left in place for two weeks, between 5 and 18 August.

**Figure 2:**
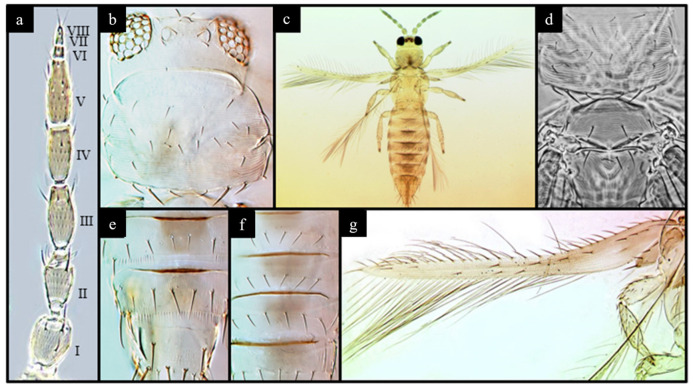
(**a**) I–VIII antennae segments; (**b**) compound eyes with ocelli and ocelli setae; (**c**) female *S. dorsalis*; (**d**) pronotum, mesonotum, and metanotum; (**e**) tergites V–VIII; (**f**) sternites V–VII; (**g**) fore wing with cilia [17].

**Figure 3:**
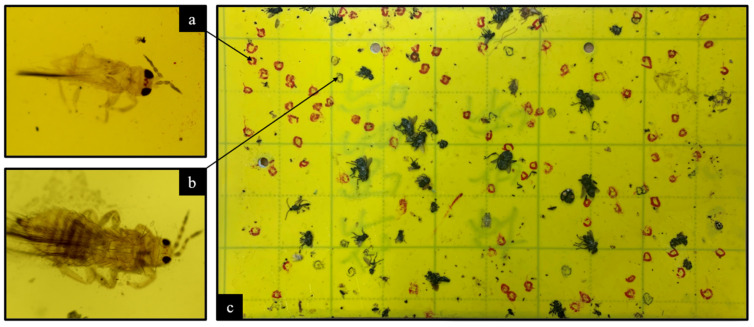
(**a**) *S. dorsalis* thrips (100× magnified); (**b**) Thrips of a species other than *S. dorsalis* (100× magnified); (**c**) the yellow sticky trap with thrips of different types marked manually with pen ink (*S. dorsalis* in red and any other thrips species in black).

Traps were gathered after being exposed and labeled by knowledgeable staff in the laboratory. The insects were closely examined under a microscope on each square millimeter to distinguish between different species of thrips, using taxonomic indices that focused on characteristics, such as the size, shape, and color of different parts of the insect, as listed in Table 1 and shown in Figure 2. The researchers were particularly interested in identifying one species of thrips among many, so they marked the position of any thrips found on the traps using two different colored ink pens according to the institute’s internal convention. Thrips belonging to the *S. dorsalis* species were marked (surrounded) in red, whereas those of any other species were marked in black, as shown in Figure 3.

**Figure 4:**
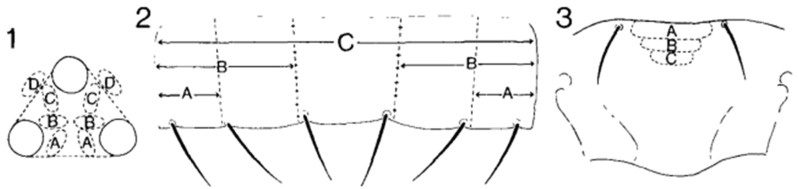
State of characters 4, 15, and 8 in Table 2; (**1**) ocellar triangle showing ocellar setae pair III position; (**2**) sternite showing distribution of microtrichia; (**3**) metanotum showing median setae position.

Great attention should be given when identifying thrips due to the high level of similarity among many species, which can lead to confusion. A notable example of this, as by A. M. Dickey et al., is the long-standing observation made between *S. dorsalis* and *Drepanothrips reuteri* [18] (Figure 4). However, upon closer examination, certain traits can aid in their differentiation. The following are some distinguishing characteristics for these two species:

**Table 2:** *Scirtothrips dorsalis* and *Drepanothrips reuteri* character distribution [19].

Species	1	2	3	4	5	6	7	8	9	10	11	12	13	14	15	16	17
*D. reuteri*	+	+	−	C	2	4	25	A	4	3–4	+	3	−	−	A	+	P, NA, SA, O
*S. dorsalis*	+	+	+	A	2	4	25–30	B	4	2	−	3	+	+	C	−	P, O, A

1. Abdominal tergites with dark median area. 2. Tergal antecostal ridge dark. 3. Sternal antecostal ridge dark. 4. Ocellar setae pair III position (Figure 4(1)). 5. Number of major postocular setae medially. 6. Number of pronotal posteromarginal setae. 7. Length of pronotal B2 setae (in Mm approx.). 8. Metanotal median setae position (Figure 4(3)). 9. Number of anteromarginal setae on forewing scale. 10. Number of second vein setae of forewing. 11. Forewing cilia wavy (+) or straight (−). 12. Number of setae on tergal microtrichial fields. 13. Microtrichia present anteromedially on tergite VIII. 14. Microtrichia present medially on tergite IX. 15. Distribution of microtrichia on sternites (Figure 4(2)). 16. Drepanae present on tergite IX of male. 17. Distribution: P = Palearctic; NA = North American; SA = South American; O = Oriental; A = Australian.

### 2.2. Imaging System

**Figure 5:**
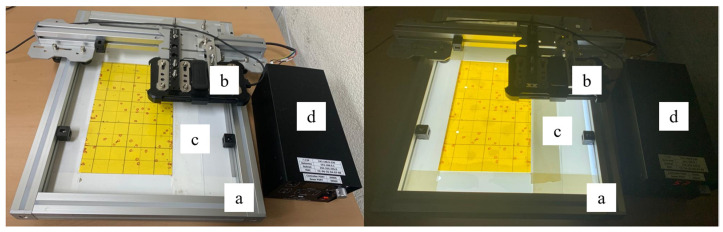
Imaging system (turned OFF on (**left**) and ON on (**right**)) in mainly 3 parts: (a) slider frame, (b) smartphone camera, (c) LED panel, (d) power controller.

To create a portable system that could be used by farmers in the field, it was necessary to collect a dataset under the same conditions as those encountered in the field. To achieve this, a portable and low-cost imaging system was developed to capture thrips images on trap cards. The system was comprised of a lightweight frame that held the smartphone camera directly above the trap card. The camera was mounted on two sliders (a frame), which allowed it to move in two dimensions and capture images of the entire trap card in a grid-like pattern, as shown in Figure 5.

#### 2.2.1. Frame

A custom-designed camera slider frame was constructed from lightweight aluminum profiles of 30 × 30 mm circumference and carefully assembled with screws in our laboratory for precision and portability, with a size of 460 × 360 mm, which matched the dimensions of the base plate, which was 5 mm thick. The longest frame arms are designed to have a furrow line along each side of the arm length, serving as rails that were connected to two pairs of wells. These wells allowed for the horizontal movement of the camera, as shown in Figure 5a.

The crossing bar was 430 mm long and an additional length compared to the 360 mm frame short arm, and was fixed each side of the rail holder plate. Additionally, the crossing bar contained rails that held two pairs of wells connected to a phone-holder supporter component. Here, the phone holder was fixed with screws, and the wells assisted in the vertical movement of the camera. Frame movements were restricted and locked in length to ensure that the camera only captured the area of the plate covered by the trap, which was 252 × 150 mm. The horizontal and vertical movements of the camera allowed the entire area of the trap card to be captured in a grid-like pattern.

The frame was lightweight and easy to disassemble, making it suitable for field use. The aluminum materials used in the frame helped reduce the overall weight of the system, making it easy to transport.

#### 2.2.2. Smartphone Camera

The imaging system employed a Samsung Galaxy S21 Ultra smartphone running on an Android operating system (Android 13, One UI 5.1) as its camera. The phone’s camera was an ultra-wide (13 mm) 12-megapixel lens with autofocus and digital zoom capabilities, featuring an f/2.2 aperture and 1/2.55” sensor size that enabled it to capture high-quality images, even in low-light environments. Its 120-degree field of view allowed for a wider area of the trap card (50 × 50 mm) to be covered when held 55 mm above the card [20]. The phone was securely attached to the camera holder of the imaging frame with a universal phone holder, which allowed for easy removal and replacement, as shown in Figure 5b.

The phone was connected to a computer running Samsung Dex [21], which is a user-friendly software program that provides a simple interface for operating the camera functions of the phone, to fine-tune the camera settings and manipulate the captured images.

#### 2.2.3. Lighting Component

After observing poor-quality images captured under normal lighting conditions, the normal lab lighting and base plate on the imaging system frame were replaced with a custom-designed LED panel (Figure 5c). This panel featured four vertically arranged LED lights with equal spacing and covered by a semi-transparent white plate to create an even lighting source. The panel was positioned at the same location as the previous base plate (30 mm thick) to accommodate the additional casing needed to house the lights. Each of the four lights was connected to a power controller via its own channel (Figure 5d), providing greater control over lighting intensity and direction. The updated lighting system offered superior illumination for imaging purposes.

An LED illuminator controller was responsible for regulating the intensity of the four lights in the LED panel, as previously described. This device was connected to each light through individual channels and provided precise control over the brightness level of each light. The controller leveraged a pulse-width modulation technique to adjust the lighting intensity, and the brightness of each light source could be adjusted independently within the range of 0 to 255. Consequently, the controller offered full control over the entire lighting coverage of the imaging system. The controller (EN-0412 model) and lighting device were produced by LWS, a company headquartered in South Korea [22].

### 2.3. Image Acquisition and Dataset Preparation

The two-directional movements of the phone camera over the trap card enabled the collection of a dataset of thrips populations, which were used to develop a deep learning thrips detection model. The images of this dataset needed to be consistently acquired as camera frame data (video streams) for the thrips detection system, which was the final product of the current project. To achieve this, systematic rules and measurements were established and followed throughout the entire system building process.

#### 2.3.1. Image Capture and Overlapping Techniques

A smartphone camera held at a height of 55 mm from the LED plate surface (i.e., the trap card) was used to capture the entire 250 × 150 mm area of the trap by capturing multiple images in a grid format. These images had a 1:1 aspect ratio and a resolution of 2992 × 2992 pixel. However, an overlapping technique during image capture was utilized to ensure that the imaging experiment resulted in comprehensive and high-quality data, and this was based on 24 preset phone positions over the trap card.

**Figure 6:**
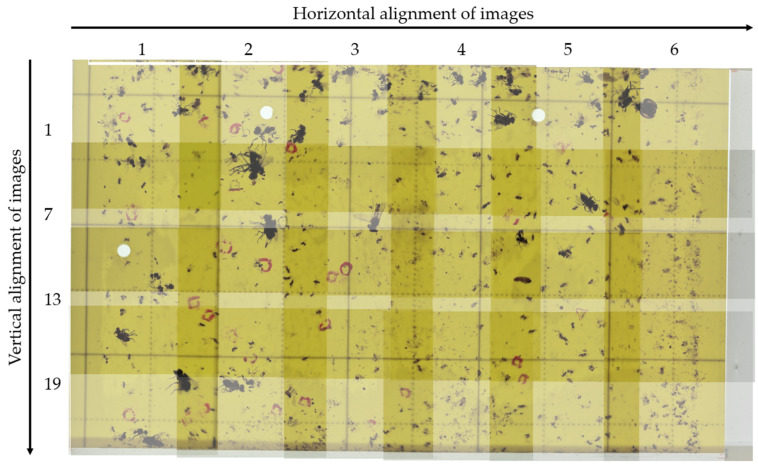
A grid of 24 images from no. 1 of the 65 used trap cards, showing image overlaps (dark areas between 2 and 4 neighbor images) and capturing directions (horizontal and vertical alignments) under transmittance lighting condition.

The proposed overlapping technique enabled the complete coverage of the target area to be captured without gaps or blind spots. The area of interest was divided into a grid of 24 images (each measuring 50 × 50 mm) that compensated for the distortions caused by the close proximity of the camera to the target area. Overlapping ensured that the distortions present in one image were corrected by adjacent overlapping images. However, the overlapping technique significantly increased the size of the dataset. Approximately 27% and 44% overlaps were necessary to ensure sufficient overlap between images for horizontal and vertical edges, respectively. This resulted in a 27–54% horizontal increase and a 44–88% vertical increase in the dataset for each image, as shown in Figure 6.

**Figure 7:**
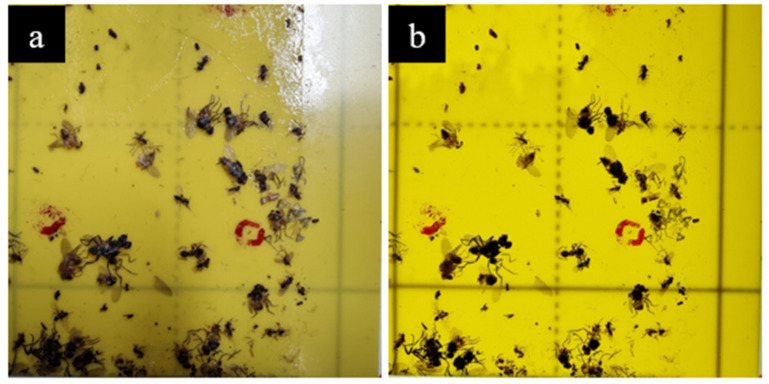
Two images captured from the same position but under different lighting conditions: (**a**) reflectance lighting and (**b**) transmittance lighting.

Two types of datasets were collected to assess the influence of lighting on the quality of the images. Two images were captured for each of the 24 camera positions over the yellow trap. First, the reflectance dataset was created under normal laboratory lighting conditions, as shown in Figure 7a. Second, the Transmittance dataset was obtained by switching on the lighting components, as described in Section 2.2.3 and shown in Figure 7b. The intensity of the lights was set to 255 for the second image capture, the maximum intensity for the setup, which was sufficient to make the insects visible.

The dataset names (reflectance and transmittance) were based on the illumination techniques. In the first technique, the camera was placed between the target and light source, so the object was visible from the reflected light and sensed by the camera. In the second technique, the target was placed between the light source and camera, so the camera visualized the target (trap card) from the light traversing it to reach the camera sensor.

### 2.4. Data Preprocessing and Image Annotation

#### 2.4.1. Image Splitting

**Figure 8:**
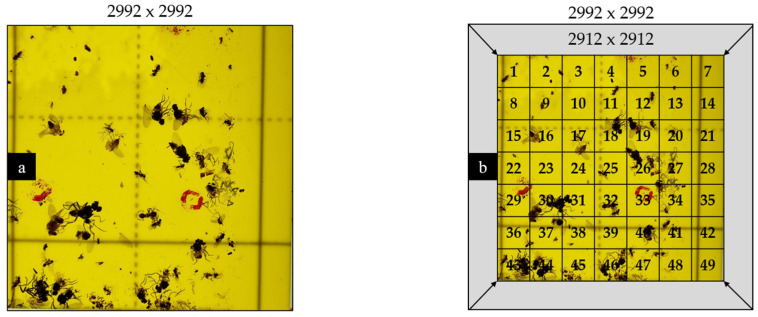
(**a**) The original image with dimensions 2992 × 2992 px, and (**b**) 49 resized images with dimensions 2912 px × 2912 px obtained by splitting the image into a grid of 7 × 7.

Our datasets images were resized to 416 × 416 pixel, as that was the maximum size for images to be used on the labeling Roboflow (an online computer vision platform we used that simplifies the process of building, deploying, and managing machine learning models for image and video analysis. It offers powerful tools for data preprocessing, annotation, and model training). However, resizing the original 2992 × 2992-pixel images in Figure 8a directly to 416 pixels would result in a loss of image quality. To address this, the images were resized to the closest multiple of 416, which was 2912 (7 × 416). Subsequently, the resulting images were split into 7 × 7 grids, with each grid cell measuring 416 × 416 pixel in resolution, as shown in Figure 8b. The resizing and splitting were performed using Python code, which allowed the quality of the images to be maintained and met the requirements of Roboflow’s labeler.

#### 2.4.2. Dataset Annotation

Roboflow’s online labeling tool was used to annotate the two datasets, transmittance and reflectance, which both contained two classes: *S. dorsalis* (SD) and other types of thrips (OT). This tool allowed bounding boxes to be drawn around the thrips in the images. The images in both datasets were superposable, meaning that two images for either dataset were captured for each camera position. Therefore, only the transmittance dataset was labeled because it had clearer images. Subsequently, the labeled data were transferred to the reflectance dataset, which contained fewer clear images. A total of 76,440 images were annotated for each dataset, which corresponded to 24 camera positions, 49 splits in 1 image (7 × 7), and 65 total traps. However, images that did not contain at least one whole thrips or its parts were filtered out, resulting in 2932 annotated images (5864 images for both datasets).

The annotation data were exported in the following two file formats after the images were labeled:

1. YOLO v5 PyTorch: A text file format with each line representing an annotated object in an image. Each line contains the class label index and the bounding box coordinates (x, y, width, height) of the object, separated by spaces.
2. TensorFlow TFRecord: A binary file format used in TensorFlow for efficient storage and retrieval of large amounts of data. It is commonly used for storing training datasets in TensorFlow, allowing for faster data loading during model training by serializing and compressing the data.

These formats were compatible with the object detection models used for analysis. Different object detection models were trained and evaluated to detect and classify thrips in the images using the annotated images and their corresponding labeling data.

#### 2.4.3. Data Splitting and Augmentation

Two datasets were utilized for the purpose of training object detection models in this study. To ensure the effectiveness of the training process, each dataset was divided into three subsets: training (70%), validation (20%), and testing (10%). Various data augmentation techniques were applied to the training dataset to improve the generalization ability of the model while avoiding the loss of objects at the edges of the images. These techniques included horizontal and vertical flipping and 90° rotations in the clockwise, counterclockwise, and upside-down directions in RoboFlow.

### 2.5. Model Training and Evaluation

Four object detection models were evaluated for the thrips detection project: EfficientDet-D0, SSD MobileNetV2, Faster R-CNN, and YOLOv5. These models were chosen because they are well known in the computer vision community [23] and have unique strengths and weaknesses to compare and explore in order to select the best model for the proposed application.

EfficientDet-D0 is a state-of-the-art model that achieves high accuracy with few computational resources [24]. SSD MobileNetV2 is fast and efficient, making it suitable for real-time applications [25,26]. Faster R-CNN is a region-based model known for its high accuracy, but can be computationally expensive [27]. YOLOv5 is a single-stage detector known for its simplicity and speed, which make it suitable for real-time applications [28].

To perform a fair comparison between the models, the thrips datasets described earlier in this document were used and trained on an NVIDIA TITAN RTX, a high-performance GPU that provided a significant advantage in terms of training time and computing power [29].

Three criteria were set for model selection:

1. The best mean average precision (mAP) at 0.5;
2. The least inference time;
3. The smallest size of the trained model files.

These criteria were chosen based on the goal of developing a thrips detection model that could run internally on a smartphone without an Internet connection. Additionally, the model required high accuracy, reasonable inference time, and a smaller file size.

The YOLO v5 PyTorch format with Pytorch v1.11.0 was used for the YOLOv5 model. TensorFlow TFRecord with TensorFlow v2.11.0 was used for the other three models to ensure a fair comparison. This decision was based on ease of implementation and the compatibility of each model with its respective format.

#### Definitions for the Selection Criteria

The definitions for the selected criteria are as follows:

1. Inference time and model size

During model inference, the inference time refers to the time taken by the model to predict the output for a given input. The inference time depends on the model’s complexity, hardware, and input size. Model size is the storage space required to store the trained model on a device or server.

**Figure 9:**
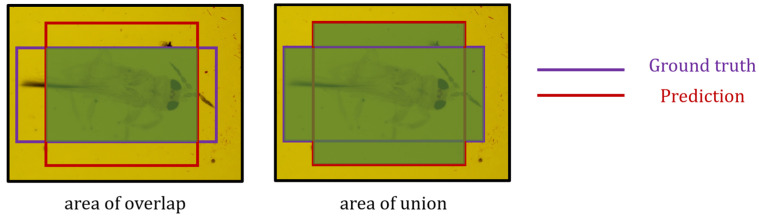
Area of overlap vs. area of union.

2. Mean Average Precision (mAP) at 0.5:

mAP is a common metric for evaluating object detection models based on the average precision at different intersection over union (IoU) thresholds. IoU measures the overlap between the predicted and actual bounding boxes (Figure 9). It was calculated as the ratio of the area of intersection of the boxes to the area of their union. To gain a deeper understanding of model performance, confusion matrices (Table 3) can be employed alongside mAP.

(1) $$\mathrm{IoU}=\frac{\mathrm{area}\;\mathrm{of}\;\mathrm{overlap}}{\mathrm{area}\;\mathrm{of}\;\mathrm{union}}$$

(2) $$\mathrm{Precision}=\frac{\mathrm{True}\;\mathrm{positives}\;\left(\mathrm{TP}\right)}{\mathrm{Total}\;\mathrm{number}\;\mathrm{of}\;\mathrm{predicted}\;\mathrm{positives}\;\left(\mathrm{TP}\;+\;\mathrm{FP}\right)}$$

(3) $$\mathrm{Recall}=\frac{\mathrm{True}\;\mathrm{positives}\;(\mathrm{TP})\;}{\mathrm{Total}\;\mathrm{number}\;\mathrm{of}\;\mathrm{actual}\;\mathrm{positives}\;(\mathrm{TP}+\mathrm{FN})}\;$$

AP (average precision) = area under precision-recall curve(4)

mAP = (AP_1 + AP_2 + … + AP_n)/n (n is the number of classes)(5)

mAP@0.5 = (AP@0.5_1 + AP@0.5_2 + … + AP@0.5_n)/n (@0.5 is the IoU threshold)(6)

### 2.6. Smartphone Application Development

The proposed smartphone application was developed using React Native, a popular open-source framework for building mobile applications [30]. The software utilizes the React Native Vision Camera plugin, which allows access to the device’s native camera and manipulation of its default settings. Specifically, the ultra-wide back camera of the Samsung S21 Ultra devices was leveraged to capture high-quality images for the real-time detection of thrips. The camera settings were modified to improve image quality, such as by adjusting the focus. A powerful and user-friendly thrips detection application was created with these features for use in the field.

## 3. Experimental Results

### 3.1. Thrips Dataset

#### 3.1.1. Manual Counting

A total of 2677 thrips were manually counted on 65 traps using an optical microscope. These thrips were classified into two types: *S. dorsalis* and other thrips. The results are presented in Table 4.

#### 3.1.2. Image Acquisition and Dataset Clearing

The raw dataset collected in the experiment was comprised of 76,440 images for each of the two lighting conditions (reflectance and transmittance). The total number of images was determined as follows:

1. Twenty-four 2992 × 2992-pixel images were captured by traps, using six horizontal and four vertical positions of the phone camera over the trap while moving on the frame sliders. The overlaps between neighboring images were considered, as described in the second paragraph of Section 2.3.1.
2. Subsequently, 7 × 7-pixel grid images were generated from these 24 images using the Open-cv Python library to satisfy the requirements of the Roboflow online labeler. First, the images were resized from 2992 × 2992 pixel to 2912 × 2912 pixel, which was the nearest multiple of 416, the required size for Roboflow to be labeled. Thus, 49 images were generated from each raw image.
3. The above steps were applied to all 65 images, and we obtained 76,440 images (65 × 24 × 49).

### 3.2. Model Evaluation

#### 3.2.1. Best Model(s) Selection

**Figure 10:**
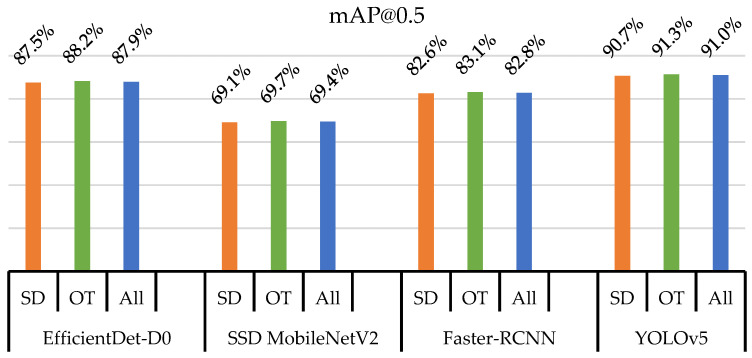
Model evaluation based on mAP at 0.5 of IoU.

The bar chart in Figure 10 shows the mAP@0.5 values for the two classes of thrips (SD and OT) and their average values (All) detected using four different object detection models: YOLOv5, EfficientDet, Faster R-CNN, and SSD MobileNetV2. The results were based on an evaluation of the models on 416 × 416-pixel images.

YOLOv5 and EfficientDet-D0 outperformed the other models in terms of accuracy, achieving average mAP@0.5 values of 91.0% and 87.9%, respectively. Faster R-CNN and SSD MobileNetV2 achieved lower accuracies, with average mAP@0.5 values of 82.8% and 69.4%, respectively. A slight difference was observed for the individual classes, where the OT class was detected more accurately than the SD class for the same model.

**Figure 11:**
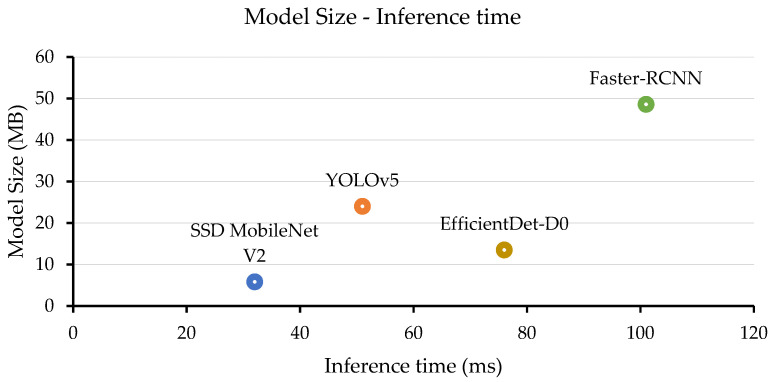
Model evaluation based on the model size and inference time.

In terms of speed, SSD MobileNetV2 was the fastest model with an average inference time of 32 ms per image, as shown in Figure 11. YOLOv5 and EfficientDet-D0 had inference times of 51 and 76 ms per image, respectively. Faster R-CNN had the slowest inference time of 101 ms per image. In terms of model size, SSD MobileNetV2 had the smallest model size of 5 MB. EfficientDet-D0 had the second smallest model size of 13.5 MB. YoLOv5 and Faster R-CNN had larger model sizes of 24 and 48.6 MB, respectively.

The results in Figure 10 and Figure 11 suggest that YOLOv5 and EfficientDet were the most accurate models for detecting thrips, whereas SSD MobileNetV2 was the fastest and most lightweight.

#### 3.2.2. Best Dataset Based on Lighting Condition

**Figure 12:**
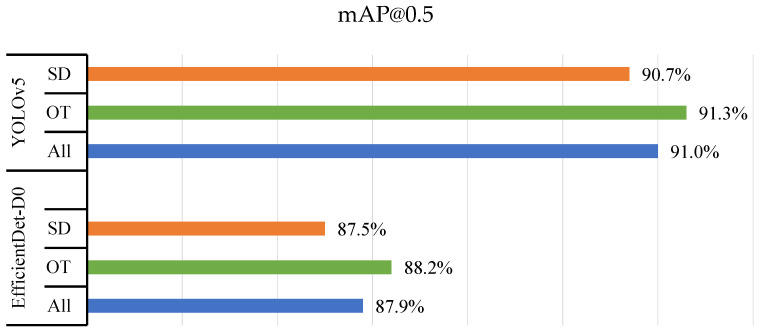
Model training using reflectance dataset.

**Figure 13:**
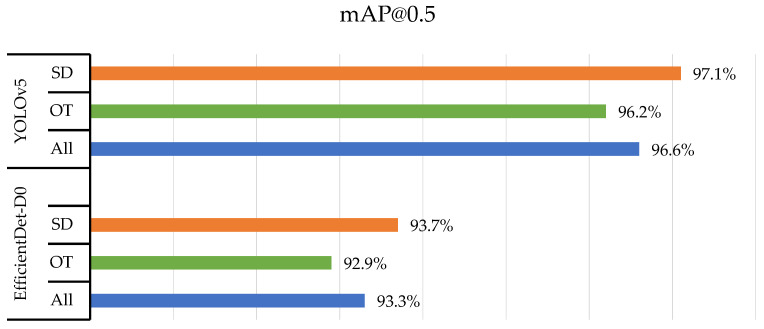
Model training using transmittance dataset.

**Figure 14:**
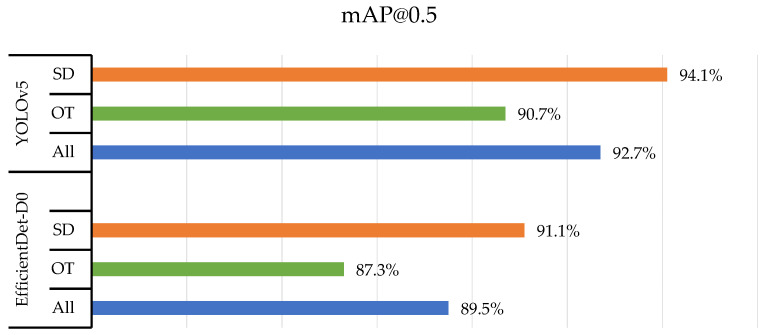
Model training using reflectance–transmittance dataset.

The following bar charts display the mAP@0.5 values for the detection of two classes of thrips (SD and OT) using the YOLOv5 and EfficientDet-D0 models on three different datasets: reflectance (2932 images—Figure 12), transmittance (2932 images—Figure 13), and reflectance + transmittance (5864 images—Figure 14).

The YOLOv5 and EfficientDet-D0 models achieved high accuracy in detecting both classes of thrips in all three datasets. YOLOv5 achieved a slightly higher mAP@0.5 value for both classes compared to EfficientDet-D0 for the reflectance dataset. YOLOv5 and EfficientDet-D0 achieved mAP@0.5 values of 90.7% and 87.5% for SD and 91.3% and 88.2% for OT, respectively. YOLOv5 outperformed EfficientDet-D0 by a greater margin in the transmittance dataset. YOLOv5 and EfficientDet-D0 achieved mAP@0.5 values of 97.1% and 96.2% for SD and 96.2% and 92.9% for OT, respectively.

When the two datasets were combined in the reflectance + transmittance dataset, YOLOv5 continued to achieve higher mAP@0.5 values for both classes of thrips. YOLOv5 and EfficientDet-D0 achieved mAP@0.5 values of 94.1% and 90.7% for SD and 92.7% and 87.3% for OT, respectively.

The compiled confusion matrix in Table 5 displays the distribution of accurately and inaccurately predicted positions of thrips among the 2548 bounding boxes labeled in the reflectance and transmittance datasets, as well as 5096 in the reflectance + transmittance dataset, for the two classes.

Overall, the results suggest that YOLOv5 was more accurate than EfficientDet-D0 in detecting thrips in all three datasets, with a higher margin of difference in the transmittance dataset. It is also worth noting that the mAP@0.5 values for both models were generally higher in the transmittance dataset than in the reflectance dataset. This may be because the transmitted images provided clearer and more distinct features for thrips detection.

### 3.3. Real-Time Thrips Detection Application

After analyzing the results of the experiments, it was concluded that the EfficientDet-D0 model trained on the transmittance dataset was the most suitable choice for the development of the proposed real-time native-based thrips detection reaction smartphone application.

Although YOLOv5 achieved a higher accuracy in all results, the EfficientDet-D0 model had a significantly smaller model size and inference time, making it more suitable for embedded devices such as smartphones. It was crucial to use a model that was optimized for speed and size to ensure fast and reliable performance of the device because the goal was to build a smartphone application that could work without an Internet connection.

The EfficientDet-D0 model trained on the transmittance dataset was the best choice for the proposed real-time thrips detection reaction-native-based smartphone application owing to its small model size, fast inference time, and good performance on the relevant dataset.

**Figure 15:**
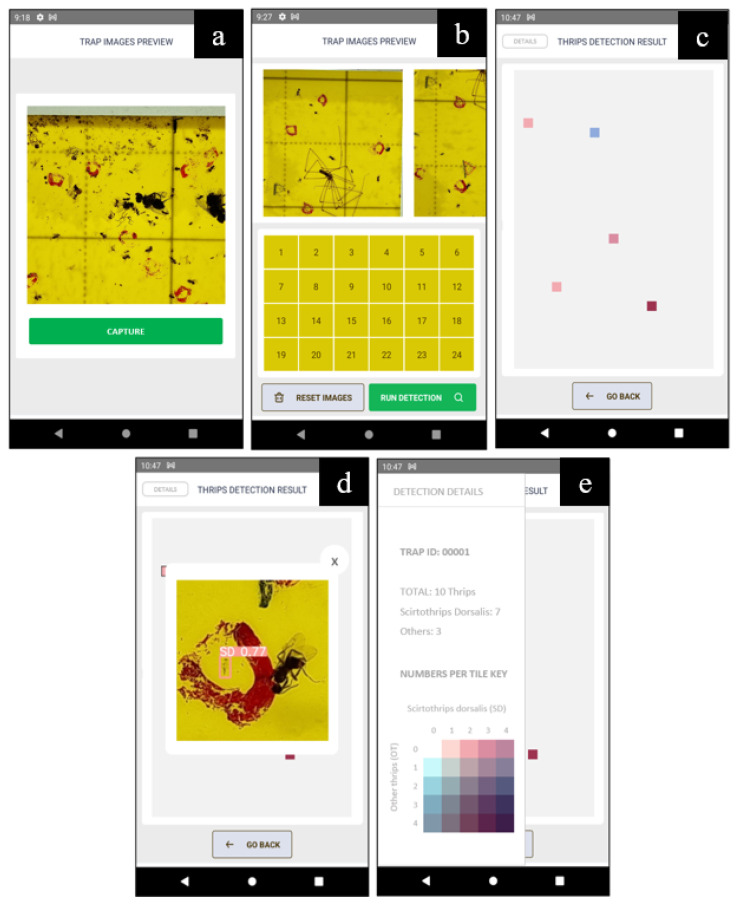
The smart phone application screen for real-time thrips detection. (**a**) Camera view of the image to be detected from. (**b**) The visualization of the captured images. (**c**) Thrips detection results map. (**d**) Automatic magnified thrips image to show the detected insect. (**e**) The trap detail panel with insect number and trap map key.

This research led to the development of a real-time object detection smartphone application that was optimized to work on the same device used for dataset image acquisition, the Samsung Galaxy S21 Ultra. The application included the TF Lite model file format, which was converted from the EfficientDet-D0-trained model on the transmittance dataset. The detection process was simple, as shown in Figure 15.

Twenty-four images from the traps were collected using the same frame as that used for dataset collection. The application accessed the ultra-wide back camera of the phone and detected the presence of thrips on the trap in real-time. The operator clicked on the CAPTURE button and the image was saved based on the current position among the 24 images, as shown in Figure 15a.

The 24 captured images are shown in Figure 15b. Subsequently, the detected thrips positions in the entire trap were visualized, as shown in Figure 15c. These positions are marked in red or blue squares, representing the presence of SD and OT, respectively. The colors are in four different intensities, from lighter to darker, meaning from one to four thrips of either class in that image tile. Color combinations may also occur when both classes are present in the same tile.

If an operator clicks on one of the colors, a magnified image of the thrips position will be displayed with insect(s), as shown in Figure 15d. If needed, the left drawer panel can be opened to show the entire contents of the thrips trap and map of color combinations with their related number of thrips, as shown in Figure 15e.

**Figure 16:**
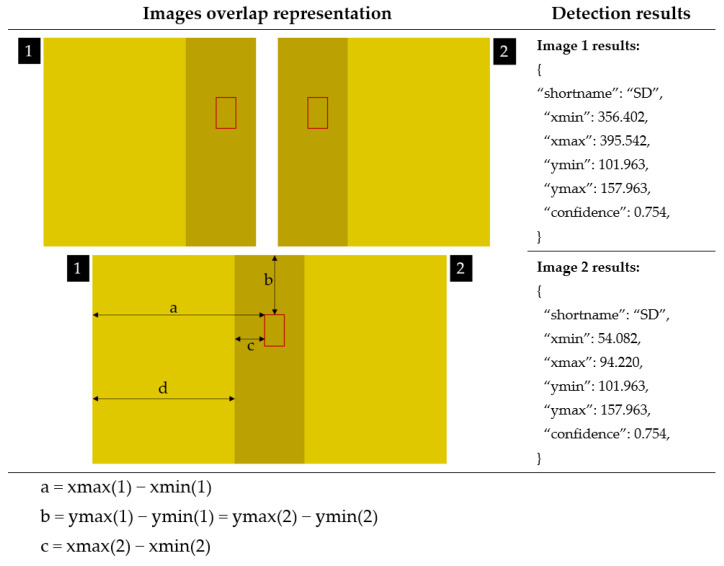
Removing bounding boxes duplication from overlapping images results generated by the detection model on 2 neighbor images (camera frames) in the smartphone application.

The counting algorithm used to generate the results was based on the total number of bounding boxes obtained from the trap and is illustrated in Figure 15e. The total was obtained from the count of all boxes for the 24 images, followed by elimination of duplications caused by overlapping neighboring images (considering the most accurate of the overlapping boxes), as illustrated in Figure 16.

The illustration in Table 3 represents a real case of image overlap, namely, b(1) = b(2) and a − d = c, where a, b, and c are the distances of the bounding box to the edges of the image. The same insect will appear in images 1 and 2 if the values for “shortname” keys are the same for the two images results.

**Figure 17:**
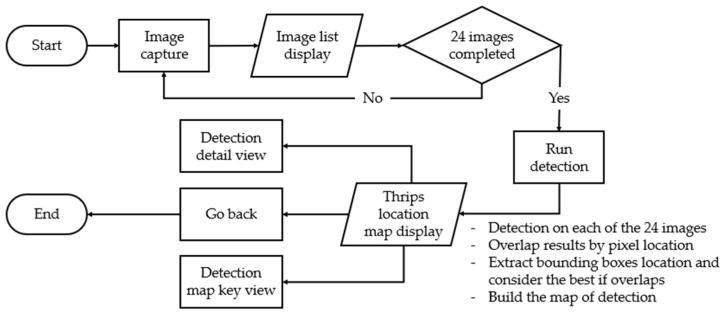
Workflow diagram for insect detection.

From start to end, the Figure 17 can illustrate the system detection process.

## 4. Discussion

The objective of this study was to create a smartphone-based deep learning system that can efficiently and effectively detect and classify *S. dorsalis*, a significant orchard citrus fruit garden pest in Jeju Island, South Korea, in real time. Our findings demonstrated that the EfficientDet-D0 model, trained on the transmittance dataset, achieved an overall accuracy of 93.3% in detecting *S. dorsalis* and other thrips on fruit crops. Particularly, the model exhibited higher accuracy in detecting *S. dorsalis* (93.7%) compared to other thrips (92.9%), highlighting its efficiency in targeting the specific species of interest.

In addition to its accuracy, our system has the potential to reduce pesticide usage by facilitating timely and targeted interventions. Early detection of pests minimizes damages and enables growers to take prompt action, reducing the need for widespread pesticide application [31] and leading to significant cost savings [32] while positively impacting the environment [33].

Compared to conventional methods, our smartphone-based system for thrips detection and classification offers several advantages. It provides a more efficient and cost-effective approach for growers, delivering real-time and accurate data on thrips populations without requiring extensive training or expertise. The system outperforms traditional methods such as the detection of adult bean thrips in navel oranges performed by J. A. Harman et al., where the results showed that a light trap captured 41.1% of thrips in a tent, while washing the thrips out of the navel resulted in close to 90% recovery using various rinses [34]. The method achieved accuracy rates of 93.9% and 89.9% for whitefly and thrips detection, respectively, with a coefficient of determination (R^2^) of 0.9785 and 0.9582 when compared to manual counting.

Additionally, it revolutionizes thrips detection in the field by being non-destructive, fast, efficient, and cost-effective. It eliminates the need for specialized equipment and trained personnel, making it easily accessible to a wide range of users, including growers, field workers, and researchers. The system’s portability and offline usability make it suitable for deployment in remote areas or places with limited internet coverage.

Traditional methods such as morphological keys, which rely on morphological features for identification, require extensive training, are time-consuming, and may not be effective for closely related species [35]. Despite their limitations, these methods remain valuable for taxonomic studies [36]. Morphometrics, an alternative method utilizing geometric measurements, offers more objectivity and reproducibility but still requires expertise and time [37]. Morphometric analyses conducted on populations of *S. dorsalis* from different continents showed significant distinctions between Asian populations and four other populations, based on two to five morphological characteristics, depending on the specific populations being compared [38]. Protein analysis, while providing a 100% accuracy rate for distinguishing *S. dorsalis* from other species, is impractical for field use due to the need for specialized equipment and expertise [39]. Molecular methods such as PCR and DNA barcoding have shown promise, with accuracy rates reaching 86% [40].

Recent research has demonstrated the potential of deep learning models for thrips detection, with accuracies comparable to our system; for instance, another computer-vision-based pest detection system, which utilizes machine vision and artificial intelligence, was developed to monitor the Asian citrus psyllid in citrus groves, showing potential for automating scouting procedures and detecting the pest with 80% precision [41]. This further confirms the potential of our system for various crop pest detection applications.

While previous studies using machine learning have achieved success in thrips detection, they often have limitations in terms of equipment and practicality. Our system overcomes these limitations by being smartphone-based and designed for real-time field detection, making it accessible to growers without the need for expensive equipment [42].

Although various methods for thrips detection exist, including trapping [43], visual inspection [44], pheromone-based methods [45], chemical traps, and sticky traps [46], they all have limitations in terms of accuracy and efficiency. Our system, utilizing controlled lighting conditions and high-quality images captured by a Samsung (Galaxy S21 Ultra) ultra-wide back camera, ensures accurate thrips detection and classification. However, it is important to acknowledge that the accuracy of the system may vary in real-world scenarios where phone cameras and image quality can be unpredictable.

To enhance the accuracy and usability of the system, several key improvements should be considered. Firstly, expanding the training dataset to include a diverse range of *S. dorsalis* specimens from different geographic locations is essential. This will enable the model to effectively handle variations in image quality and environmental conditions encountered in different farming regions.

Addressing the challenge of false positives and false negatives is crucial. Despite achieving a high accuracy rate, there is still a possibility of misidentifying pests. Therefore, integrating the system with other pest management strategies will provide a more comprehensive approach to pest control, reducing the impact of misclassifications.

Continued research and development efforts are necessary to build robust models that can handle varying image qualities encountered in real-world farm settings. Regular updates to the system are vital to ensure adaptability to changing crop conditions, environmental factors, and thrips populations. Additionally, considering the evolving nature of smartphone and phone camera technologies, ongoing device support improvements should be implemented. Furthermore, incorporating language adaptability in the system would enable interpretation by farmers worldwide, facilitating its widespread adoption and usability. Lastly, expanding the system’s capabilities to detect and identify thrips species beyond *S. dorsalis* would provide a more comprehensive pest management solution.

Despite these limitations, our system offers a more accurate and efficient approach to thrips detection compared to traditional methods. It has the potential to revolutionize pest management in fruit crops by providing growers with a user-friendly, cost-effective, and accessible tool for early detection and classification of thrips. By minimizing pesticide usage and enabling targeted interventions, our system contributes to sustainable agricultural practices while safeguarding crop yields.

## 5. Conclusions

This study developed a real-time thrips detection smartphone application optimized for working on embedded devices, such as smartphones, without the need for an Internet connection. The performances of two state-of-the-art object detection models (YOLOv5 and EfficientDet-D0) were compared using three different datasets (reflectance, transmittance, and reflectance + transmittance) to detect two classes of thrips (SD and OT). The results indicated that YOLOv5 achieved higher accuracy in all the datasets. However, EfficientDet-D0 with the transmittance dataset was the most suitable choice for the proposed real-time thrips detection application because of its smaller model size, fast inference time, and reasonable performance on the relevant dataset. The developed application used the EfficientDet-D0 model trained on the transmittance dataset to detect and visualize the presence of thrips in real-time, making it a valuable tool for the monitoring and management of thrips in agriculture.

Overall, this research demonstrates the potential of using computer vision and deep-learning techniques for pest detection and monitoring in precision agriculture, with practical implications for pest control and crop yield improvement. Future research should focus on improving the accuracy and efficiency of the developed application, exploring the use of other machine learning models and datasets, and extending its application to other types of pests and crops.

## Figures and Tables

**Table 1:** Some of distinctive characteristics of *Scirtothrips dorsalis* [17].

Body Part	Characteristics
Wings	Fully winged in both sexes; forewings usually strongly shaded but paler toward apex.
Color (female)	Yellow with brown marking medially on tergites III–VII; sternites without brown markings.
Antennae	Segment I pale, II shaded, III–VIII dark.
Head	About twice as wide as long; postocular and ocellar region closely striate.
Eyes	Compound eyes with no ommatidia strongly pigmented.
Ocelli	Posterior ocelli with ocellar setae pair III arising between them, well behind tangent between anterior margins.
Pronotum	Closely striate; postero-marginal setae S2 30–35 microns and clearly longer than S1.
Metanotum	Sculpture variable, usually transversely arcuate anteriorly with irregular longitudinal reticulations or striations posteriorly.
Fore Wing	First vein with three setae on distal half, and scale with four marginal setae; second vein with two (or three) setae; postero-marginal fringe cilia all straight.
Tergites	The III–V with bases of median setae usually closer together than length of these setae; tergal microtrichial fields with three discal setae; VIII with discal microtrichia present anteromedially, and postero-marginal comb complete.
Sternites	Microtrichia extending across median area on posterior half.
Male Characteristics	Smaller in size than female; tergite IX without drepanae, and aedeagus apparently with no armature.

**Table 3:** Confusion matrix table.

	Predicted Positive	Predicted Negative
**Actual Positive**	True Positive (TP)	False Negative (FN)
**Actual Negative**	False Positive (FP)	True Negative (TN)

**Table 4:** Distribution of 2677 in 2370 SD (*S. dorsalis*), and 307 OT (other thrips) on 65 traps.

No.	SD	OT		No.	SD	OT		No.	SD	OT		No.	SD	OT		No.	SD	OT
**1**	28	2		**14**	74	1		**27**	36	14		**40**	7	2		**53**	3	0
**2**	22	12		**15**	32	23		**28**	28	16		**41**	12	2		**54**	43	2
**3**	6	1		**16**	13	4		**29**	15	18		**42**	10	1		**55**	12	0
**4**	14	4		**17**	14	6		**30**	11	2		**43**	7	5		**56**	56	1
**5**	36	1		**18**	36	22		**31**	6	6		**44**	8	0		**57**	34	1
**6**	25	16		**19**	5	20		**32**	4	2		**45**	115	2		**58**	131	0
**7**	131	4		**20**	17	3		**33**	23	13		**46**	24	1		**59**	5	2
**8**	50	0		**21**	22	8		**34**	24	13		**47**	10	0		**60**	12	1
**9**	196	4		**22**	165	0		**35**	26	7		**48**	25	0		**61**	53	1
**10**	23	3		**23**	16	1		**36**	18	5		**49**	89	15		**62**	6	0
**11**	57	3		**24**	1	0		**37**	20	8		**50**	12	2		**63**	125	0
**12**	9	0		**25**	58	2		**38**	41	16		**51**	1	0		**64**	2	0
**13**	68	4		**26**	16	2		**39**	1	1		**52**	111	0		**65**	70	2

**Table 5:** Compiled confusion matrix for the two chosen models on three datasets (validation sets).

Dataset Type	Model		Predicted SD		Predicted OT	
Reflectance (2548 bounding boxes)	YOLOv5	Actual SD ^1^	2035	TP ^3^	209	FN ^4^
		Actual OT ^2^	26	FN	278	TP
	EfficientDet-D0	Actual SD	1964	TP	280	FN
		Actual OT	36	FN	268	TP
Transmittance (2548 bounding boxes)	YOLOv5	Actual SD	2179	TP	65	FN
		Actual OT	12	FN	292	TP
	EfficientDet-D0	Actual SD	2103	TP	141	FN
		Actual OT	22	FN	282	TP
Reflectance + transmittance (5096 bounding boxes)	YOLOv5	Actual SD	4223	TP	265	FN
		Actual OT	57	FN	551	TP
	EfficientDet-D0	Actual SD	4089	TP	399	FN
		Actual OT	77	FN	531	TP

^1^ Scirtothrips dorsalis, ^2^ other thrips, ^3^ True Positive, ^4^ False Negative.

## Data Availability

Data is contained within the article.
